# Characterization of *Clostridium novyi* isolated from a sow in a sudden death case in Korea

**DOI:** 10.1186/s12917-020-02349-9

**Published:** 2020-05-06

**Authors:** Chang-Gi Jeong, Byoung-Joo Seo, Salik Nazki, Byung Kwon Jung, Amina Khatun, Myeon-Sik Yang, Seung-Chai Kim, Sang-Hyun Noh, Jae-Ho Shin, Bumseok Kim, Won-Il Kim

**Affiliations:** 1 College of Veterinary Medicine, Jeonbuk National University, 79 Gobong-ro, Iksan, Jeonbuk 54596 Republic of Korea; 2 School of Applied Biosciences, College of Agriculture and Life Sciences, Kyungpook National University, Daegu, 41566 Republic of Korea; 3 Department of Pathology, Faculty of Animal Science and Veterinary Medicine, Sher-e-Bangla Agricultural University, Sher-e-Bangla Nagar, Dhaka, 1207 Bangladesh; 4 MSD Animal Health Korea Ltd., Seoul, 04637 Republic of Korea

**Keywords:** Anaerobe, *Clostridium novyi*, Isolation, Characterization, Whole-genome sequencing

## Abstract

**Background:**

Multifocal spherical nonstaining cavities and gram-positive, rod-shaped, and endospore-forming bacteria were found in the liver of a sow that died suddenly. *Clostridium novyi* type B was identified and isolated from the sudden death case, and the isolate was characterized by molecular analyses and bioassays in the current study.

**Results:**

*C. novyi* was isolated from the liver of a sow that died suddenly and was confirmed as *C. novyi* type B by differential PCR. The *C. novyi* isolate fermented glucose and maltose and demonstrated lecithinase activity, and the cell-free culture supernatant of the *C. novyi* isolate exhibited cytotoxicity toward Vero cells, demonstrating that the isolate produces toxins. In addition, whole-genome sequencing of the *C. novyi* isolate was performed, and the complete sequences of the chromosome (2.29 Mbp) and two plasmids (134 and 68 kbp) were identified for the first time. Based on genome annotation, 7 genes were identified as glycosyltransferases, which are known as alpha toxins; 23 genes were found to be related to sporulation; 12 genes were found to be related to germination; and 20 genes were found to be related to chemotaxis.

**Conclusion:**

*C. novyi* type B was isolated from a sow in a sudden death case and confirmed by biochemical and molecular characterization. Various virulence-associated genes were identified for the first time based on whole-genome sequencing.

## Background

*Clostridium novyi* (*C. novyi*), originally named *Bacillus oedematis maligni* no. 2, was first isolated in 1894 from guinea pigs by Dr. Frederick Novy [1]. *C. novyi* is broadly distributed in soil, water and marine sediments and affects humans and animals worldwide [2–4]. *C. novyi* is a gram-positive, noncapsulated, motile obligatory anaerobe that produces endospores to resist unfavorable environments [1, 5].

Based on the toxins they produce, *C. novyi* are classified into four types: A, B, C and D. *C. novyi* type A produces alpha, gamma, delta and epsilon toxins. *C. novyi* type B produces alpha, beta, and zeta toxins, while type C produces gamma toxin [2, 6, 7]. *C. novyi* type D is considered to be a different species, *Clostridium haemolyticum*, because it does not produce alpha toxin and because the disease that it causes is different from those caused by types A and B [8]. *C. haemolyticum* produces beta, eta and theta toxins [9]. *C. novyi* type A is frequently involved in gas gangrene infections in humans and animals, while type B is the etiological agent of infectious necrotic hepatitis (black disease), which is typically observed in sheep, cattle and swine [10]. *C. novyi* type C is not known to induce illness in and is typically considered nonpathogenic toward laboratory animals [7]. *C. novyi* type D (*C. haemolyticum*) is responsible for hemoglobinuria in calves [7], while *C. novyi* types A and B (producing alpha toxin) cause sudden death in swine, and the carcasses exhibit gross distension and livers with gas bubble infiltration or sponge-like appearances [11, 12].

The 16S rRNA gene sequence has been used to detect genetic relatedness between different species of bacteria. Currently, next-generation sequencing is utilized as a rapid tool to perform whole-genome sequencing of clinical isolates. Indeed, this method has proved to be of great value for understanding bacterial evolution, outbreaks, toxigenicity, and antimicrobial resistance in a number of studies involving *Vibrio cholera, Escherichia coli, Clostridium difficile,* and *Mycobacterium tuberculosis* [12, 13]. In the present study, for the first time, *C. novyi* type B was isolated from a sudden death case of a sow in Korea, and the isolate was characterized by molecular analyses and bioassays. To the best of our knowledge, this is the first report of the complete genome sequence of *C. novyi* type B in Korea.

## Results

### Isolation of the *C. novyi* isolate

**Fig. 1:**
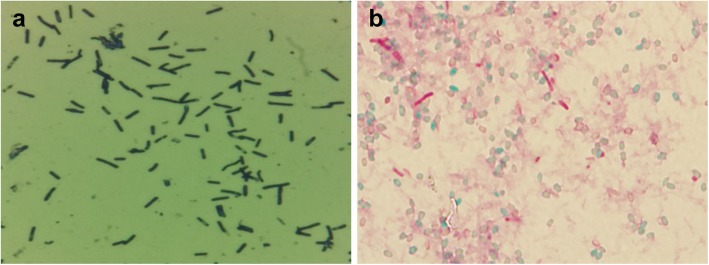
Morphology of the *Clostridium* isolate based on staining. **a** Gram staining was performed on the cultured isolate, resulting in the detection of gram-positive rods. **b** Endospores of the cultured *C. novyi* isolate were observed after staining with 5% malachite green

After 72 h of anaerobic incubation, colonies showing irregular shapes with unclear borders appeared on agar media, and gram-positive, rod-shaped, endospore-forming bacteria were identified in the colonies (Fig. 1a and b). Differential PCR was conducted on the DNA extracted from a single colony, which confirmed the isolate as *C. novyi* type B [14]. No other bacterial and viral pathogens were detected (data not shown).

### Biochemical characterization of the *C. novyi* isolate

Biochemical analysis revealed that the *C. novyi* isolate generates gas when treated with hydrogen peroxide and exhibits beta hemolysis on blood agar. The biochemical characteristics of the *C. novyi* isolate are summarized in Table 1. The *C. novyi* isolate was positive for D-glucose, gelatin, D-maltose, salicin, L-rhamnose, D-cellobiose, and lecithinase and negative for L-tryptophan, urea, D-mannitol, D-lactose, D-saccharose, D-xylose, L-arabinose, esculin, glycerol, D-mannose, D-melezitose, D-sorbitol, D-trehalose, and catalase.

**Table 1:** Biochemical test results for the *C. novyi* isolate

Active ingredients	Results
D-Glucose	+
Gelatin	+
Lecithinase	+
L-Tryptophan	–
Urea	–
D-Mannitol	–
D-Lactose	–
D-Saccharose	+
D-Maltose	+
Salicin	+
D-Xylose	–
L-Arabinose	–
Esculin	–
Glycerol	–
D-Cellobiose	+
D-Mannose	–
D-Melezitose	–
D-Sorbitol	–
L-Rhamnose	+
D-Trehalose	–
Catalase	–
Endospore	+
Gram	+
Hemolysis	+

+ positive; − negative

### Phylogenetic analysis of the isolate based on the 16S rRNA gene sequence

**Fig. 2:**
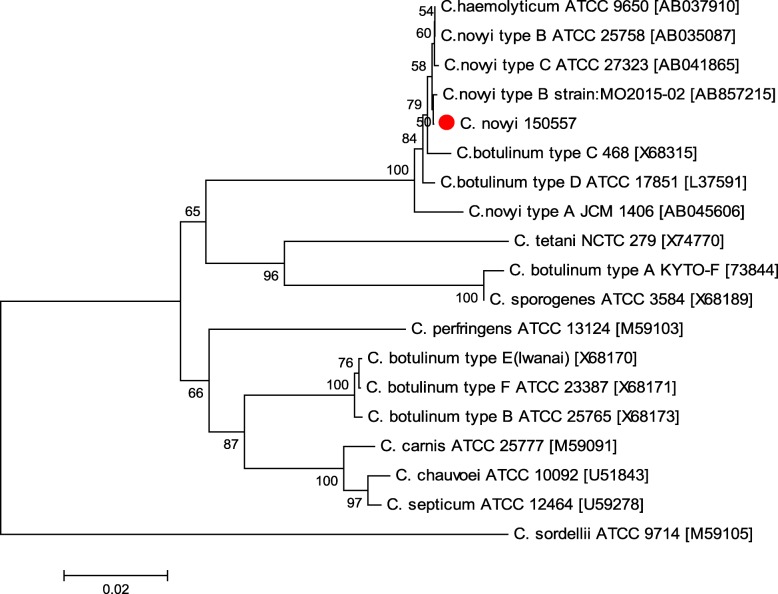
Phylogenetic analysis of the 16S rRNA gene sequences of *Clostridium* spp. The tree was constructed using the neighbor-joining method with 1000 replicates for bootstrap values with MEGA 6. The scale bar represents 0.02 substitutions per nucleotide position. The circle indicates the isolate identified in this study

Based on the 16S rRNA gene sequence analysis, the *C. novyi* isolate showed more than 99% similarity with *C. novyi* types B and C, *C. haemolyticum*, and *C. botulinum* types C and D. The *C. novyi* isolate exhibited 84 to 91% similarity with *C. perfringens*, *C. sporogenes*, and *C. sordellii*. A comparison of the 16S rRNA gene sequences of the *C. novyi* isolate with those of different *Clostridium* species is shown in Fig. 2.

### Histopathological examination of the liver

**Fig. 3:**
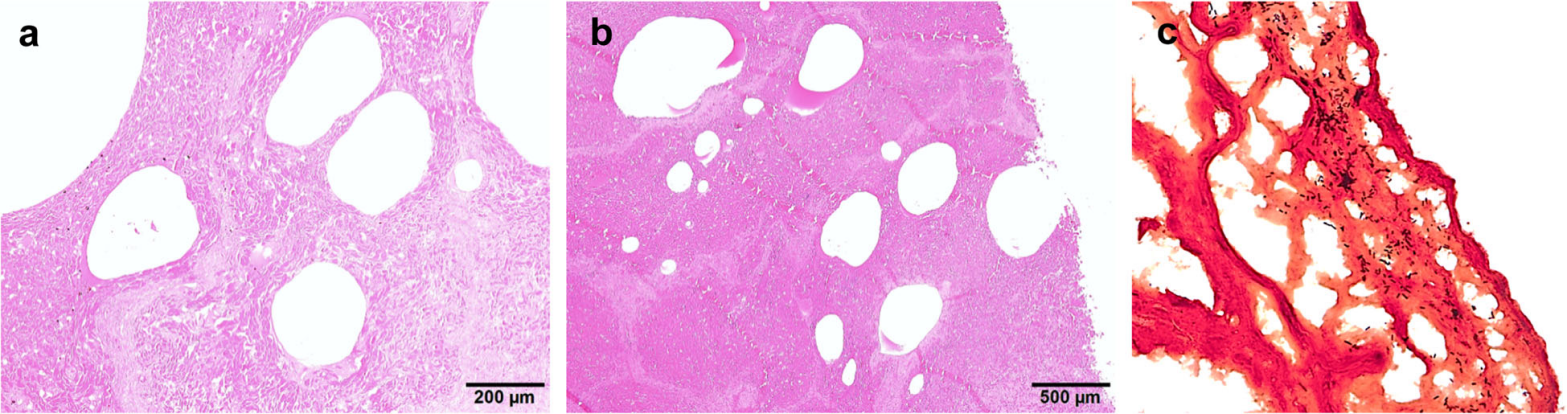
Morphology of the *Clostridium* isolate based on staining. **a** and **b** Coagulation necrosis and multifocal spherical non-staining cavities were detected on tissue section. (**c**) Gram staining was performed on liver tissue (400×)

The liver tissues showed coagulative necrosis throughout the entire field. Multifocal spherical nonstaining cavities were found that were well demarcated, and the cavity margins were clear (Fig. 3a and b) Furthermore, gram-positive, rod-shaped bacteria were observed on the liver tissue (Fig. 3c).

### Cell culture assay of the *C. novyi* isolate on Vero cells

**Fig. 4:**
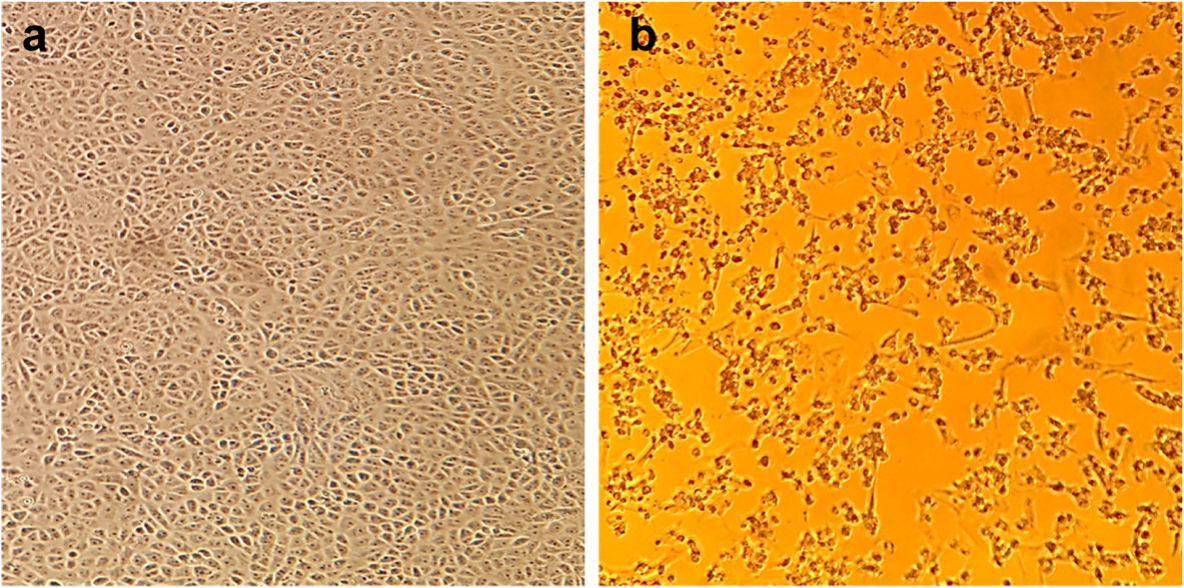
Cytopathic effect of the cell-free supernatant of the *C. novyi* isolate in Vero 76 cells. Morphological alterations of Vero 76 cells exposed to cell-free supernatant of the *C. novyi* isolate for 5 days. **a** Vero 76 cells without treatment; the cells show a fibroblast-like morphology. **b** Vero 76 cells inoculated with filtered (0.2 μm) *C. novyi* supernatant (1:32). A cytopathic effect (rounding and disintegration of cells) was observed in cells treated with *C. novyi* supernatant

The cell-free supernatant of *C. novyi* reference strains, ATCC 17861 (*C. novyi* type A) and ATCC 25758 (*C. novyi* type B) were tested on Vero 76 cells that were observed for 5 days. Abnormal cells were detected in all of the strains at days 1 and 2 for the 1:4 and 1:8 dilutions, respectively. On day 3, rounding and retracting cells were observed up to dilutions of 1:32 from inoculation of cell-free supernatants of ATCC 17861 and ATCC 25758, while the cell-free supernatant of the *C. novyi* isolate (150775) showed abnormal cells up to dilutions of 1:16. After day 4, all tested wells that were inoculated with the cell-free supernatant of *C. novyi* strains exhibited rounding and retracting cells up to the 1:32 dilutions. No cytopathic effect was observed in the Vero cell controls. Vero 76 cells with or without cytopathic effect are shown in Fig. 4.

### Sequencing and genome features of the *C. novyi* isolate

**Fig. 5:**
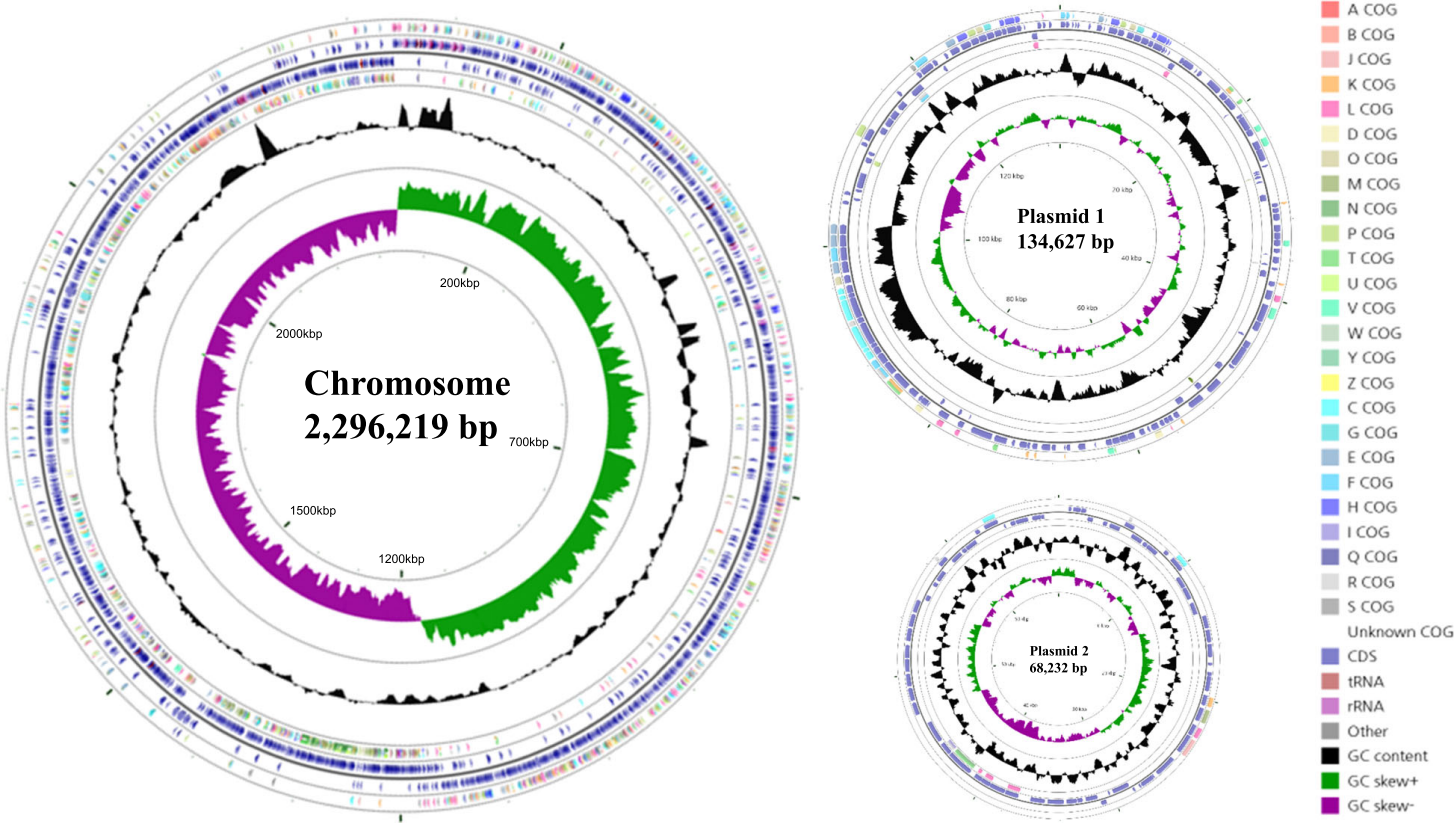
Circular map of the chromosome and two plasmids of the *C. novyi* isolate. From the outside to the center: genes on the forward strand (colored by Clusters of Orthologous Genes (COG) categories), coding DNA sequence (CDS) on the forward strand, CDS on the reverse strand, genes on the reverse strand (colored by COG categories), GC content, and GC skew

The complete genome features of the *C. novyi* isolate are summarized in Table 2 and Fig. 5. The genome consisted of a single circular chromosome with two circular plasmids. The sizes of the *C. novyi* isolate chromosome and plasmids 1 and 2 were 2,296,219 bp, 134,627 bp, 68,232 bp, respectively. The GC content of the chromosomal DNA was 27.9%, and 33 rRNA genes, 84 tRNA genes, and 54 pseudogenes were identified. The GC content of plasmid 1 was 26.3%, and 13 pseudogenes were identified. The GC content of plasmid 2 was 25.5%, and 13 pseudogenes were identified. The assembled and annotated sequences of the *C. novyi* isolate chromosome and plasmids 1 and 2 were submitted to NCBI (accession numbers: CP029458.1, CP029459.1, and CP029460.1).

**Table 2:** Genomic characteristic of the *C. novyi* isolate

	Chromosome	Plasmid 1	Plasmid 2
Genome size (bp)	2,296,219	134,627	68,232
GC content (%)	27.9	26.3	25.5
Protein coding genes	2009	141	54
rRNAs	33	–	–
tRNAs	84	–	–
Pseudogenes	54	13	13

### Prediction of genes associated with pathogenicity

All of the identified genes of the *C. novyi* isolate are summarized in Additional file 1. A total of 35 genes related to sporulation and germination were detected in the whole genome of the *C. novyi* isolate. Among these genes, sporulation-related sigma factors (DFH04_RS02880, DFH04_RS08440, DFH04_RS08465, DFH04_RS08470, and DFH04_RS10365) and stage-specific sporulation genes were detected. Additionally, 7 glycosyltransferase genes associated with the monoglycosyltransferase activity of alpha toxin were identified (DFH04_RS05070, DFH04_RS05155, DFH04_RS10400, DFH04_RS10410, DFH04_RS10425, DFH04_RS10435, and DFH04_RS10440). Various genes encoding chemotaxis proteins were also identified in the *C. novyi* isolate genome, including a sensor kinase, the product of DFH04_RS10060; a deamidase, the product of DFH04_RS08070; a methyltransferase, the product of DFH04_RS10065; a docking protein, the product of DFH04_RS08070; and a phosphatase protein, the product of DFH04_RS10055. Additionally, 9 genes were identified as methyl-accepting chemotaxis proteins (MCPs).

## Discussion

The isolation and identification of *C. novyi* is rarely successful because specimens must be delivered to laboratories very quickly under strict anaerobic conditions [12, 13, 15]. In addition, samples should be processed within 12 h of the death of pigs for accurate diagnosis and successful isolation of *C. novyi* before the proliferation of other bacteria in the carcass [11, 12, 16]. In this study, postmortem examination of a sow sudden death case was conducted, and a liver sample was collected within approximately 11 h. After extracting DNA from the sample, the presence of *C. novyi* in the sample was confirmed by differential PCR.

Biochemical assays of the isolated colonies showed the presence of lecithinase activity and glucose and maltose fermentation, which are the characteristic features of *C. novyi*. Previously, lecithinase activity and glucose and maltose fermentation have been associated with *C. novyi* types A and B [17]. On the basis of biochemical tests, our isolate could also be differentiated from other closely related Clostridia, such as *C. haemolyticum,* which lacks the ability to ferment maltose, while *C. botulinum* types C and D display variable maltose fermentation capabilities and maltose and lecithinase activity [17].

The 16S rRNA gene sequencing results for the *C. novyi* isolate showed that the isolate shared approximately 98–99% similarity with each type of *C. novyi*, *C. haemolyticum* and *C. botulinum* types C and D. In particular, the 16S rRNA gene sequence of the *C. novyi* isolate and that of *C. haemolyticum* showed the highest similarity (99.6%). Consistent with this finding, high similarity (> 99.9%) between the 16S rRNA gene sequence of *C. novyi* type B and that of *C. haemolyticum* has been reported in a previous study [18]. Therefore, different methods are needed to distinguish *C. novyi* type B from *C. haemolyticum*. The N- and C-terminal amino acid sequences of FliC are well preserved between *C. novyi* type B and *C. haemolyticum*, but the central region amino acid sequences are not [19]. Therefore, the *C. novyi* isolate was distinguished from *C. haemolyticum* using *fliC* gene primers.

In this study, virulence-associated genes were identified by whole-genome sequencing of the *C. novyi* isolate. The other identified genes are summarized in Additional file 1. Endospores are important contributors to pathogenesis before being the vegetative forms of *C. novyi* type B. Furthermore, the endospores of *C. novyi* are highly resistant to environmental conditions. *Clostridium spp.* initiate the sporulation process when unfavorable conditions are detected. The sporulation process is a carefully orchestrated cascade of events at both the transcriptional and posttranslational levels that involves a multitude of sigma factors, transcription factors, proteases, and phosphatases. *Clostridium spp.* genomes contain genes for all major sporulation-specific transcription and sigma factors. The sporulation process consists of several stages. Sporulation-specific sigma factors affect each sporulation stage [20]. Sporulation genes and sigma factor genes were detected in the *C. novyi* isolate; in addition, the *C. novyi* isolate was found to contain germination genes that initiate the germination process when a favorable environment is detected. Although the mechanism has not been completely elucidated, after *C. novyi* endospores are ingested, they are absorbed from the intestine and reach the liver via the portal circulation. Subsequently, the endospores are spread to other organs. The endospores germinate and produce toxins in organs when anaerobic conditions form [15].

The main pathogenic protein of *C. novyi* type B is alpha toxin. Alpha toxin is produced and released by the vegetative forms of *C. novyi* type B, and its monoglycosyltransferase activity inactivates several GTP-binding proteins in cells, resulting in modification and redistribution of the actin cytoskeleton. For these reasons, necrosis occurs in the liver, and the cut surface of the affected liver exhibits a sponge-like appearance [15, 21–25]. In this study, glycosyltransferase genes were detected in the genome of the *C. novyi* isolate. In addition, the effect of the toxins produced by the isolate was tested by observing the effect of the cell-free supernatant of the *C. novyi* isolate on Vero cells, although the alpha toxin of the isolate was not purified. The cytopathic effect produced was similar to the observations in a previous study in which the purified alpha toxin exhibited a strong effect on Vero cells, resulting in rounded cells in addition to lysed cells [26]. Histologically, multifocal spherical non-staining cavities were detected in tissue sections, and gram-positive rods were observed in the liver tissue by Gram staining. These findings were consistent with the results of previous studies [11, 12, 15, 16]. Based on above results, the *C. novyi* isolate was considered to have the capability of producing alpha toxin toward on Vero cells and liver tissue.

Chemotaxis enables bacteria to move according to chemical gradients. Chemotaxis affords key physiological benefits, including enhanced contact with growth substrates. Another important aspect of chemotaxis is that it plays a role in infection and disease, as chemotaxis signaling pathways are widely distributed among diverse pathogenic bacteria [27]. In this study, 15 genes related to chemotaxis, namely, DFH04_RS10060 (CheA), DFH04_RS10080 (CheW), DFH04_RS10075 (CheD), DFH04_RS08070 (CheV), DFH04_RS10055 (CheC), DFH04_RS10065 (CheR), and nine MCPs were detected in the genome of the *C. novyi* isolate. As reported previously [27], the products of these genes transfer signals by phosphorylation and activate the flagellum. The activated flagellum enables the bacterium to move toward an attractant. Chemotaxis-associated genes were also detected in a previous study based on *Clostridium novyi*-NT, which is an attenuated strain of *C. novyi* [28].

## Conclusion

This *C. novyi* isolate was first isolated from a sudden death case of sow in Korea and confirmed by biochemical and molecular characterization. Furthermore, various virulence-associated genes were identified in the genome of the isolate, indicating that the isolate might have had a role in the sudden death of the sow. However, more research including animal experiments is needed to determine the pathogenicity of *C. novyi*. Nonetheless, the complete genomic sequence of *C. novyi* isolate will contribute to a better understanding of the biology of *C. novyi* and related species.

## Methods

### Sample collection and isolation of *C. novyi*

Diagnostic samples, primarily livers, collected from sows with sudden death within 12 h after death were submitted to the Jeonbuk National University Veterinary Diagnostic Center in 2015 (case no. 150557) [14]. Swab samples taken from affected sites on the livers were inoculated in Reinforced clostridial medium (RCM) (BD Biosciences, New Jersey, USA) and incubated anaerobically at 37 °C for 72 h in a gas jar. Then, the incubated broth medium was heated at 100 °C for 10 min, reinoculated in 5% sheep blood agar, and again incubated under the same conditions. Colonies suspected of being *C. novyi* that exhibited beta hemolysis on sheep blood agar plates were subsequently identified as *C. novyi* type B based on differential PCR, Gram staining and 16S rRNA gene sequencing [14].

The morphology of the bacteria in the identified colonies was further confirmed using endospore staining. Spores and vegetative cells were identified using a 5% malachite green staining solution (Thermo Fisher Scientific Inc., MA, USA) and a safranin staining solution (Gram stain kit solution), respectively, as described previously [29].

### DNA extraction from isolates

DNA was extracted from colonies grown on 5% sheep blood agar for differential PCR and 16S rRNA gene sequencing by Patho Gene-spin™ (iNtRON Biotechnology, Seongnam, Korea), according to the manufacturer’s instructions.

### Differential PCR for detection of the *C. novyi* isolate

The suspected colonies were confirmed using *C. novyi* specific flagellin gene (*filC*) primers, where *fliC* was amplified using differential primers for *C. novyi* types A and B, as well as *C. haemolyticum*, which is genetically close to *C. noyvi* [14, 19].

The PCR assays in this study were performed in a SimpliAmp Thermal Cycler (Applied Biosystems, Foster City, CA, USA) in a 20 μl reaction containing: 1× PCR buffer; 2 mM MgCl_2_; 250 μM each dNTP; 1 unit of DNA polymerase (AccuPower Multiplex PCR kit; Bioneer Inc., Alameda, CA, USA); and 10 pmol of each primer. The PCR conditions were as follows: initial denaturation at 94 °C for 5 min, followed by 30 cycles of denaturation at 94 °C for 30 s, annealing at 56.5 °C for 1 min and extension at 72 °C for 1 min, with a final extension at 72 °C for 5 min. The amplified PCR products were separated by electrophoresis in 2% (w/v) agarose gels and stained with Red Safe™ (iNtRON Biotechnology, Seongnam, Korea). The strains ATCC 17861 (*C. novyi* type A; 472 bp), ATCC 25758 (*C. novyi* type B; 551 bp), and KCTC 5570 (*C. haemolyticum*; 819 bp) were used as positive controls.

### Histopathological examination of the liver

Approximately 2-cm^3^ liver tissue samples were fixed in 10% phosphate-buffered formalin, routinely processed, and embedded in paraffin. Tissue sections (4 μm) were prepared using a microtome (HM-340E, Thermo Fisher Scientific Inc., MA, USA), and the sections were placed onto glass slides. Hematoxylin and eosin (H&E) staining was performed according to standard techniques, and Gram staining was performed on liver tissue collected from the dead sow using a Gram stain kit according to the manufacturer’s instructions.

### Identification and characterization of the *C. novyi* isolate

The biochemical characteristics of the *C. novyi* isolate were determined using an API 20A kit (BioMerieux, USA) according to the manufacturer’s instructions. The lecithinase activity of the *C. novyi* isolate was confirmed on an egg yolk agar plate (Kisan Biotech. Co., Seoul, Korea), where the isolate was inoculated onto the plate using a sterilized loop and incubated anaerobically for 72 h at 37 °C. The presence of opalescence around a colony was taken to indicate lecithinase activity. Additionally, a catalase test was performed using pure *C. novyi* isolates smeared on a microscope slide. A drop of 3% hydrogen peroxide (Wako, Osaka, Japan) was added, and the production of copious bubbles was taken to indicate that the bacteria were positive for catalase.

### 16S rRNA gene sequencing analysis

16S rRNA gene sequencing was performed by a sequencing facility (Biofact Co., Daejun, Korea) using a pure culture of the *C. novyi* isolate. The 16S rRNA gene sequence of the *C. novyi* isolate was identified by a BLAST search and compared with other *Clostridium* species sequences downloaded from the NCBI database. Multiple alignment of the 16S rRNA gene sequences was performed using MegAlign (DNASTAR, Madison, Wisconsin, USA), and a phylogenetic tree was constructed with the neighbor joining method using MEGA 6 [30].

### Cell culture assay of the *C. novyi* isolate on Vero cells

Vero 76 cells (ATCC CRL-1587) were maintained in high-glucose Dulbecco’s modified Eagle’s medium (DMEM; Welgene, Korea) supplemented with heat-inactivated 5% fetal bovine serum (FBS; Invitrogen, USA), 2 mM L-glutamine, and a 100× antibiotic-antimycotic solution [Anti-Anti, Invitrogen; 1× solution contains 100 IU/ml penicillin, 100 μg/ml streptomycin, and 0.25 μg/ml Fungizone® (amphotericin B)] at 37 °C under a humidified 5% CO_2_ atmosphere.

The cell culture assay was performed in Vero 76 cells as described previously [26, 31]. Briefly, *C. novyi* cell-free supernatant obtained after centrifugation was prepared by filtrating the broth through a 0.2-μm cellulose acetate syringe filter (Corning, Germany). Two-fold serial dilutions of the filtered supernatant were prepared with cell medium for up to 8 dilutions. Cell culture assays were performed in 96-well tissue culture plates (Falcon, NY, USA). Subsequently, the supernatant of cultured Vero 76 cells was discarded, and the cells were washed twice with 1× PBS. Then, the diluted cell-free supernatant of the *C. novyi* isolate was added to the Vero 76 cells, which were then incubated at 37 °C under a humidified atmosphere with 5% CO_2_ for 5 days. The cell-free culture supernatants of reference strains of *C. novyi* type A (ATCC 17861) and *C. novyi* type B (ATCC25758) were used as positive controls.

### Whole-genome sequencing

DNA was extracted from cell pellets of *C. novyi* isolate from 200-ml cultures using Patho Gene-spin™, according to the manufacturer’s instructions. A sample of high-quality, high-molecular-weight DNA was used to prepare size-selected SMRTbell templates of approximately 20 kb. A NanoDrop spectrophotometer (Thermo Fisher Scientific Inc., MA, USA) and a Qubit fluorometer (Thermo Fisher Scientific Inc., MA, USA) were used to measure the concentration of gDNA, and the sample passed quality control (QC) screening criteria (≥200 ng). For PacBio RS sequencing, 8 g of input gDNA was used for the 20-kb library preparation. For gDNA where the size range was less than 17 kb, we used a Bioanalyzer 2100 (Agilent Technologies, CA, USA) to determine the actual size distribution. If the apparent size of the gDNA was greater than 40 kb, the gDNA was sheared with a g-TUBE (Covaris Inc., Woburn, MA, USA) to produce library fragments in the optimal size range and purified using AMPure PB magnetic beads (Beckman Coulter Inc., CA, USA). Then, the concentration of the gDNA was measured using both a NanoDrop spectrophotometer and a Qubit fluorometer, and approximately 200 ng/μl gDNA was run on a field-inversion gel. A library was prepared in a total volume of 10 μl using a PacBio DNA Template Prep Kit 1.0 (for 3–10 kb), and SMRTbell templates were annealed using a PacBio DNA/Polymerase Binding Kit P6. A PacBio DNA Sequencing Kit 4.0 and 1 SMRT Cell was used for sequencing. SMRT Cells (Pacific Biosciences, CA, USA) with C4 chemistry were used, and 240-min movies were captured for each SMRT cell using the PacBio RS II (Pacific Biosciences, CA, USA) sequencing platform. The subsequent steps were based on the PacBio Sample Net-Shared Protocol, which is available at http://pacificbiosciences.com/.

### Bioinformatics analysis

All runs were performed with diffusion-based loading and analyzed using standard primary data analysis methods by implementing HGAP and Quiver [32]. The coding DNA sequences were predicted with Prokaryotic Genome Annotation Pipeline version 4.5 on the NCBI website (https://www.ncbi.nlm.nih.gov/genome/annotation_prok/). Additional functional annotation was performed with the Rapid Annotation using Subsystem Technology server [33].

## Supplementary information

**Additional file 1. **The identified genes of the *C. novyi* isolate.

## Data Availability

All data generated or analyzed during this study are included in this published article and in Additional file 1. Genome sequences obtained in the current study are deposited in GenBank under the accession numbers CP029458.1, CP029459.1, and CP029460.1. The datasets used and/or analyzed during the current study are available from the corresponding author upon reasonable request.

## Abbreviations

MCPs: Methyl-accepting chemotaxis proteins
GTP: Guanosine 5′-triphosphate
RCM: Reinforced clostridial medium
NCBI: National center for biotechnology information
CPE: Cytopathic effects
HGAP: Hierarchical genome assembly process
gDNA: Genomic DNA
